# Comparison of Gait Symmetry and Joint Moments in Unilateral and Bilateral Hip Osteoarthritis Patients and Healthy Controls

**DOI:** 10.3389/fbioe.2021.756460

**Published:** 2021-11-04

**Authors:** S. van Drongelen, S. Braun, F. Stief, A. Meurer

**Affiliations:** ^1^ Dr. Rolf M. Schwiete Research Unit for Osteoarthritis, Department of Orthopedics (Friedrichsheim), University Hospital Frankfurt, Goethe University Frankfurt, Frankfurt, Germany; ^2^ Department of Orthopedics (Friedrichsheim), University Hospital Frankfurt, Goethe University Frankfurt, Frankfurt, Germany

**Keywords:** hip osteoarthritis (hip OA), unilateral, bilateral, gait symmetry, joint load

## Abstract

Patients with unilateral hip osteoarthritis show a characteristic gait pattern in which they unload the affected leg and overload the unaffected leg. Information on the gait characteristics of patients with bilateral hip osteoarthritis is very limited. The main purposes of this study were to investigate whether the gait pattern of both legs of patients with bilateral hip osteoarthritis deviates from healthy controls and whether bilateral hip osteoarthritis patients show a more symmetrical joint load compared to unilateral hip osteoarthritis patients. In this prospective study, 26 patients with bilateral hip osteoarthritis, 26 patients with unilateral hip osteoarthritis and 26 healthy controls were included. The three groups were matched for gender, age and walking speed. Patients were scheduled for a unilateral total hip arthroplasty on the more affected/more painful side. All participants underwent a three-dimensional gait analysis. Gait kinematics and gait kinetics of patients and controls were compared using Statistical Parametric Mapping. Corrected for speed, the gait kinematics and kinetics of both legs of patients with bilateral hip osteoarthritis differed from healthy controls. Bilateral patients had symmetrical knee joint loading, in contrast to the asymmetrical knee joint loading in unilateral hip osteoarthritis patients. The ipsilateral leg of the bilateral patients could be included in studies in addition to unilateral hip osteoarthritis patients as no differences were found. Although patients with bilateral hip osteoarthritis show more symmetrical frontal plane knee joint moments, a pathological external knee adduction moment in the second half of stance was present in the ipsilateral leg in patients with unilateral and bilateral hip osteoarthritis. The lateral adjustment of the knee adduction moment may initiate or accelerate progression of degenerative changes in the lateral compartment of the knee.

## Introduction

Patients with unilateral hip osteoarthritis (OA) show a characteristic asymmetric gait pattern in which they unload the affected leg (Schmidt et al., 2017; Wesseling et al., 2018; van Drongelen et al., 2020a). By doing so patients transfer load to the knee and hip joint of the contralateral leg. Schmidt et al. (Schmidt et al., 2017) found that the peak external knee adduction moment in the second half of stance and the peak external hip adduction moment during the first half of stance were respectively 23 and 12% higher in the contralateral leg. Large cohort studies have shown that unilateral hip OA patients have an increased risk for developing knee OA, in both the ipsilateral but especially in the contralateral knee (Gillam et al., 2013; Jungmann et al., 2015; Joseph et al., 2016). The external knee adduction moment is a commonly used surrogate measure for joint loading and has been related to the initiation and progression of knee OA (Miyazaki et al., 2002; Andriacchi and Mündermann, 2006). Less frequently studied and thus less certain, is the association between the external hip adduction moment and hip OA. Hurwitz et al. (Hurwitz et al., 2001) showed that the initiation of hip OA relates to increased joint loads during walking. Here, the external hip adduction moment has been identified as a major determinant for joint loading (Lenaerts et al., 2009; Wesseling et al., 2015).

To confirm the suspect and define the degree of OA, radiologic images are used. A study of Günther and colleagues (Günther et al., 1998) found radiographic evidence of contralateral hip OA in 82.1% of patients scheduled for unilateral total hip arthroplasty (THA). Only half of these patients reported pain in the contralateral hip joint. Further, it is known that 16–35% of patients undergoing THA will receive a contralateral THA within 1 year of the initial hip replacement (Morcos et al., 2018). A recent study from the Osteoarthritis Initiative (Santana et al., 2020) found lower values for the incidence of contralateral THA after the initial THA (8%) but a clear higher incidence of total knee arthroplasty after an initial THA (32%).

Studies performing gait analysis predominantly included patients with unilateral hip OA (Shakoor et al., 2011; Allison et al., 2018; Stief et al., 2018; van Drongelen et al., 2021) and as such, studies on the gait characteristics of patients with bilateral hip OA are very limited. Berman et al. (Berman et al., 1991) only studied the spatio-temporal gait parameters. A study from Kubota et al. (Kubota et al., 2007) included kinematics and kinetics, whereas Solomonow-Avnon et al. (Solomonow-Avnon et al., 2016) recently reported on a method to reduce the hip joint reaction force by means of footwear. The study from Kubota et al. (Kubota et al., 2007) compared bilateral hip OA patients to healthy controls walking at a free and slow walking speed to eliminate the effect of speed. However, the very low walking speed makes a comparison to the literature difficult. Further, in the study of Kubota et al. (Kubota et al., 2007), no direct comparison to unilateral hip OA patients was made and no knee loads were reported.

Whether bilateral hip OA patients, scheduled for THA surgery on the more affected side, show the characteristic asymmetric gait pattern like unilateral patients or a more symmetrical gait pattern has not been studied yet. The main aim of this study was to determine the gait kinematics, gait kinetics and gait symmetry in patients with bilateral hip OA in comparison to unilateral hip OA patients and healthy controls. It was hypothesized that the gait pattern of both legs of patients with bilateral hip OA deviates from healthy controls, whereas only the affected leg of the unilateral hip OA patients deviates from the healthy control group. We further hypothesized that, in contrast to patients with unilateral hip OA, the external knee and hip adduction moments of the contralateral side are not different compared to the ipsilateral side (more affected side) in bilateral affected patients with hip OA. As a result, bilateral hip OA patients show a more symmetrical joint load compared to unilateral hip OA patients.

## Methods

### Participants

In this prospective study, data of 26 patients with bilateral hip OA and 26 gender, age and walking speed matched patients with unilateral hip OA were included. All patients were scheduled for a unilateral THA on the more affected/more painful side. Furthermore, 26 healthy controls, also matched for gender, age and walking speed, were selected from our reference database and included for comparison (Table 1). Healthy controls were included if they had no history of orthopedic surgeries, chronic or neuromuscular diseases or recent injuries to the lower extremities. None of the enrolled healthy controls reported ankle, knee, hip or back pain at the time of measurement. All patients and healthy controls gave written informed consent prior to participation in the original studies. Our institution’s medical ethics committee approved the studies under the numbers 319/11, 122/14 and 497/15.

**TABLE 1:** Anthropometric data and walking speed of patients and healthy controls.

	Bilateral patients	Unilateral patients	Healthy controls	*p*-value
Age (years)	64.5 (10.8)	64.1 (8.4)	63.3 (7.9)	0.885
Height (m)	1.70 (0.09)	1.68 (0.09)	1.68 (0.10)	0.614
Weight (kg)	77.4 (17.7)	78.4 (14.0)	69.3 (12.8)	0.063
BMI (kgm^−2^)	26.6 (4.4)	27.6 (4.2)^a^	24.6 (3.1)	0.026
Speed (ms^−1^)	0.99 (0.13)	1.01 (0.13)	1.06 (0.13)	0.177

Values are mean values with standard deviation in parenthesis.

BMI: body mass index.

a Significantly different compared to healthy controls (*p* = 0.024 with Bonferroni posthoc test).

### Gait Analysis

Patients underwent a three-dimensional gait analysis in the week before surgery, whereas the healthy controls visited the gait laboratory within the framework of a reference measurement. The healthy controls were asked to walk at a walking speed of approximately 1.0 ms^−1^, comparable to patients shortly before surgery (Schmidt et al., 2017). Kinematic data were collected using 8 Vicon MX T10 cameras (VICON Motion Systems, Oxford, United Kingdom) operating at 200 Hz and two AMTI force plates (Advanced Mechanical Technology Inc., Watertown, MA, United States) were used to synchronously collect ground reaction forces at 1000 Hz. A marker set with markers on the medial malleolus, medial femoral condyle and greater trochanter (Stief et al., 2013) additional to the standardized Plug-in-Gait marker set (Kadaba et al., 1990) was used.

Vicon Nexus (version 2.10) was used to process the marker trajectories. The resulting marker trajectories were filtered using a Woltring filter with a mean squared error setting of 10 (Woltring, 1986). The Plug-in-Gait model (VICON Motion Systems, Oxford, United Kingdom) is Vicon’s implementation of the Conventional Gait Model and is still widely used in the clinical gait analysis community (Baker et al., 2018). This model was used to calculate the kinematic and kinetic gait variables. The hip joint centers were obtained according to a geometrical prediction method using regression equations (Davis et al., 1991) whereas the centers of rotation for the ankle and knee joints were defined statically as the midpoint between the medial and lateral malleolus and femoral condyle markers. Kinematic outputs were calculated from the embedded coordinate system information. The joint angles mainly describe the orientation of the distal segment with respect to that of the proximal segment. The orientation of the pelvis is output as segment angles with respect to the laboratory-based axis system, as is the transverse plane alignment of the foot (the so-called foot progression angle). Inverse dynamics were used to estimate joint moments from force plate measurements of the ground reaction, an estimate of segment accelerations from kinematic data and estimates of segment inertial parameters (Kadaba et al., 1990; Davis et al., 1991).

### Data Analysis

For all patients the leg to be operated on (the more affected/more painful side) was considered the ipsilateral leg, whereas the non-affected side (unilateral patients) and the less affected side (bilateral patients) was called the contralateral leg. For the healthy controls the kinematics and kinetics of the left side were chosen randomly for comparison.

Kinematic and kinetic data were exported to Matlab (version R2020b, The Mathworks Inc., Natick, MA, United States) to normalize the data over the gait cycle and average the parameters over five good trials.

The following kinematic gait parameters, which are typically influenced by hip OA, were included for further analysis: thorax tilt and lean (the lateral displacement of the trunk relative to the supporting limb), pelvic tilt and obliquity, hip flexion-extension, knee flexion-extension, ankle dorsiflexion-plantarflexion and the foot progression angle (the angle of the long axis of the foot segment relative to the direction of walking). External ankle, knee and hip joint moments (expressed in Nmkg^−1^) were defined as kinetic outcomes. In addition, the impulse for the knee adduction and hip adduction moment (area under the curve (Herger et al., 2019)) were calculated.

For the peak external knee and hip adduction moment during the first and second half of the stance phase as well as for the impulse of the adduction moments, an absolute symmetry index (ASI) was determined (Robinson et al., 1987; Bosch and Rosenbaum, 2010) by the following equation:

$$ASI=\;\left|\frac{2\left(X_{L}-\;X_{R}\right)}{\left(X_{L}+\;X_{R}\right)}\right|\;\times 100\%$$

X_L_ and X_R_ are the parameters of the ipsilateral and contralateral leg for patients and the left and right leg for the healthy controls. An ASI of 0 indicates perfect symmetry.

### Statistical Analysis

Shapiro-Wilk tests were used to test for normal distribution of the anthropometrics. Since the data showed a normal distribution, one-way ANOVA (group) with Bonferroni posthoc tests were used to determine statistical differences between the anthropometrics and walking speed of the patient groups and healthy controls.

The selected gait kinematics and kinetics were evaluated using Statistical Parametric Mapping (SPM). SPM originates from the Random Field Theory (Adler and Taylor, 2007) and has been validated for 1D biomechanical data by Pataky et al. (Pataky et al., 2013). For the kinematics and kinetics, normal distribution was tested and confirmed by SPM. The SPM analyses were performed in MATLAB using the open-source spm1d code (version M.0.4.3) with a general critical threshold (*p*-value) of α = 0.05. SPM paired sample t-tests were used to compare the ipsilateral and contralateral legs of the patients. Data of the bilateral, unilateral and healthy controls were compared first with an SPM ANOVA and afterwards with independent two-sample t-tests (critical *p*-value corrected for multiple testing, α = 0.05/3). Data were considered significantly different when the waveform exceeded this critical threshold for more than four successive time points, i.e., at least 4% of the gait cycle (Wesseling et al., 2018).

Symmetry between patients and healthy controls was tested with Kruskal-Wallis tests and Mann-Whitney tests as the data appeared not be to be normally distributed. *P*-values ≤ 0.05 were considered significant for all analyses.

## Results

### Participants

In all groups (bilateral patients, unilateral patients and healthy controls), 16 women and 10 men were included. As patients and healthy controls were age and speed matched, no significant differences were present in age and speed, nor in weight and height (Table 1). Only the Body Mass Index (BMI) was significantly higher in unilateral hip OA patients compared to healthy controls. All bilateral patients had a Kellgren-Lawrence score (Kellgren and Lawrence, 1957) of at least 5 in both legs (Grade 3), whereas the unilateral patients were selected not to have a score exceeding 2 (Grade 1) in the contralateral leg. All patients reported significant pain in the ipsilateral hip joint: 34 patients scored 45.9 (11.4) on the pain section of the Hip disability and Osteoarthritis Outcome Score (Nilsdotter et al., 2003; Blasimann et al., 2014), whereas 18 patients from another study scored 55.2 (12.7) on the Harris Hip Score (Harris, 1969).

### Kinematics and Kinetics

In the ANOVA significant differences were detected between the bilateral patients, unilateral patients and healthy controls for both the ipsilateral and contralateral leg for all parameters except for the foot progression angle (not displayed) and the external ankle dorsiflexion/plantarflexion moment (Supplementary Figure 1 and Supplementary Figure 2).

**FIGURE 1:**
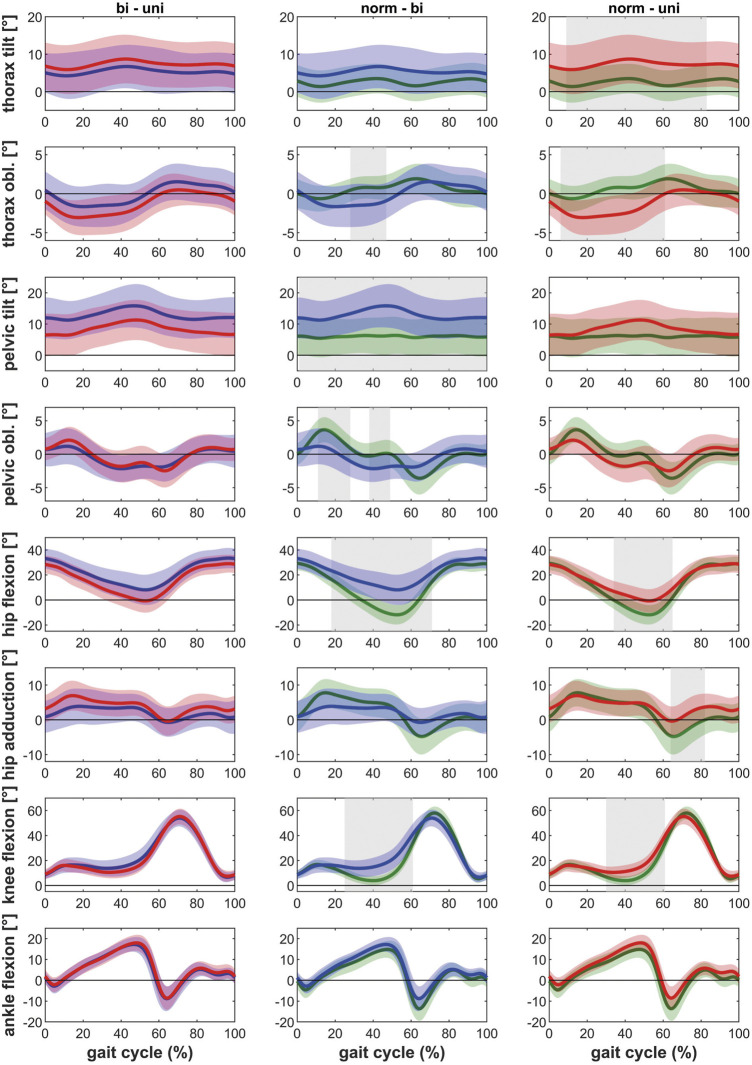
Comparison of the kinematics of the ipsilateral leg between bilateral hip OA patients, unilateral hip OA patients and healthy controls. Mean and one standard deviation of the joint angles. Shaded areas indicate statistical differences. Blue lines = bilateral patients, red lines = unilateral patients, green lines = healthy controls. **(A–C)** thorax tilt: anterior (+)/posterior (−); **(D–F)** thorax obliquity: towards the contralateral side (+)/towards the ipsilateral side (−); **(G–I)** pelvic tilt: anterior (+)/posterior (−); **(J–L)** pelvic obliquity: up (+)/down (−); **(M–O)** hip flexion/extension: flexion (+)/extension (−); **(P–R)** hip adduction/abduction: adduction (+)/abduction (−); **(S–U)** knee flexion/extension: flexion (+)/extension (−); **(V–X)** ankle dorsiflexion/plantarflexion: dorsiflexion (+)/plantarflexion (−).

**FIGURE 2:**
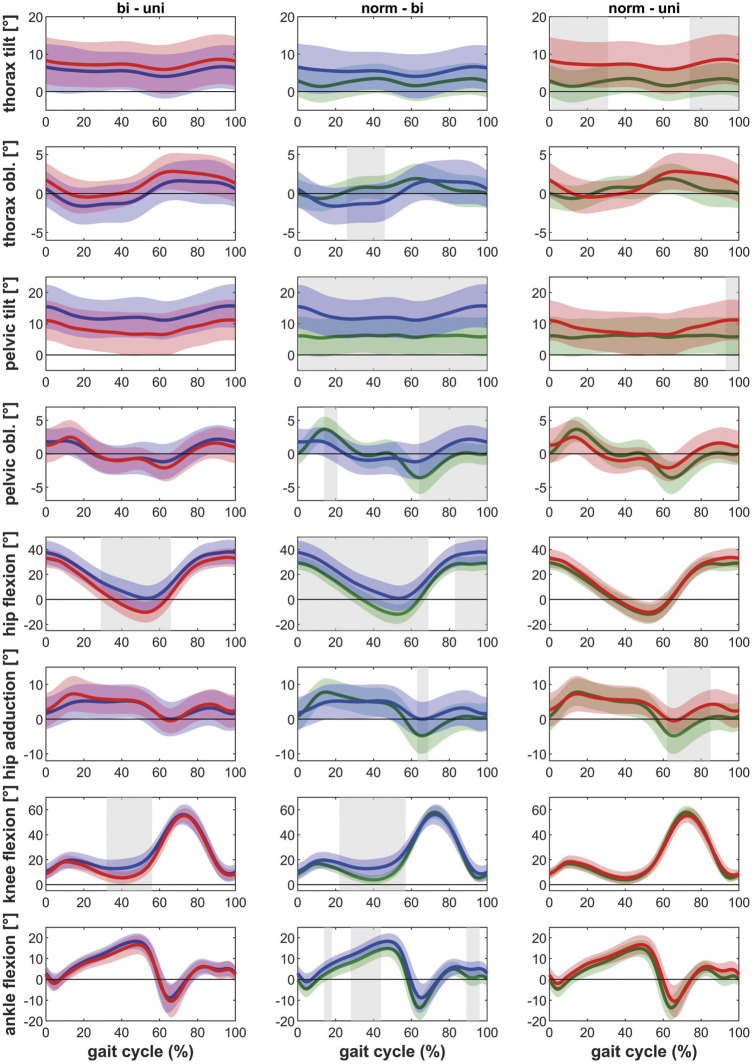
Comparison of the kinematics of the contralateral leg between bilateral hip OA patients, unilateral hip OA patients and healthy controls. Mean and one standard deviation of the joint angles. Shaded areas indicate statistical differences. Blue lines = bilateral patients, red lines = unilateral patients, green lines = healthy controls. **(A–C)** thorax tilt: anterior (+)/posterior (−); **(D–F)** thorax obliquity: towards the contralateral side (+)/towards the ipsilateral side (−); **(G–I)** pelvic tilt: anterior (+)/posterior (−); **(J–L)** pelvic obliquity: up (+)/down (−); **(M–O)** hip flexion/extension: flexion (+)/extension (−); **(P–R)** hip adduction/abduction: adduction (+)/abduction (−); **(S–U)** knee flexion/extension: flexion (+)/extension (−); **(V–X)** ankle dorsiflexion/plantarflexion: dorsiflexion (+)/plantarflexion (−).

Posthoc tests showed that bilateral patients walked with more thorax lean towards the ipsilateral side, more pelvic tilt and less pelvic obliquity up, less hip extension as well as less knee extension during the stance phase of gait compared to healthy controls (Figure 1). The contralateral leg showed additionally less hip abduction during the swing phase of gait and more dorsiflexion in the ankle joint (Figure 2). The range of motion of both the hip and the knee are limited in the bilateral hip OA patients.

Unilateral patients showed a similar pattern as the bilateral patients for the ipsilateral leg compared to the healthy controls, with more thorax lean towards the ipsilateral side and less hip and knee extension (Figure 1). However also differences were detected: whereas the bilateral patients had less pelvic obliquity up and more pelvic tilt, the unilateral patients showed more thorax tilt and less hip abduction (swing phase). For the contralateral leg, the unilateral patients only showed more thorax tilt and less hip abduction in the swing phase compared to healthy controls (Figure 2).

The ipsilateral leg of bilateral patients did not show any differences compared to the ipsilateral leg of unilateral patients. The contralateral leg of bilateral patients showed less hip and knee extension compared to the contralateral leg of unilateral patients (Figure 1 and Figure 2).

**FIGURE 3:**
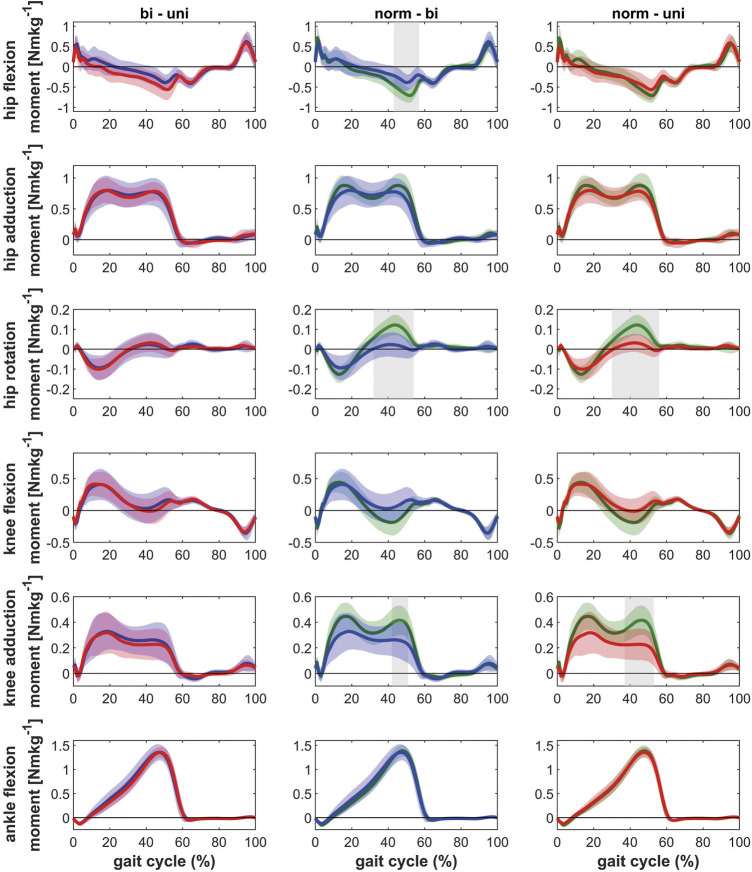
Comparison of the kinetics of the ipsilateral leg between bilateral hip OA patients, unilateral hip OA patients and healthy controls. Mean and one standard deviation of the external joint moments. Shaded areas indicate statistical differences. Blue lines = bilateral patients, red lines = unilateral patients, green lines = healthy controls. **(A–C)** hip flexion/extension moment: flexion (+)/extension (−); **(D–F)** hip adduction/abduction moment: adduction (+)/abduction (−); **(G–I)** hip rotation moment: internal rotation (+)/external rotation (−); **(J–L)** knee flexion/extension moment: flexion (+)/extension (−); **(M–O)** knee adduction/abduction moment: varus (+)/valgus (−); **(P–R)** ankle dorsiflexion/plantarflexion moment: dorsiflexion (+)/plantarflexion (−).

**FIGURE 4:**
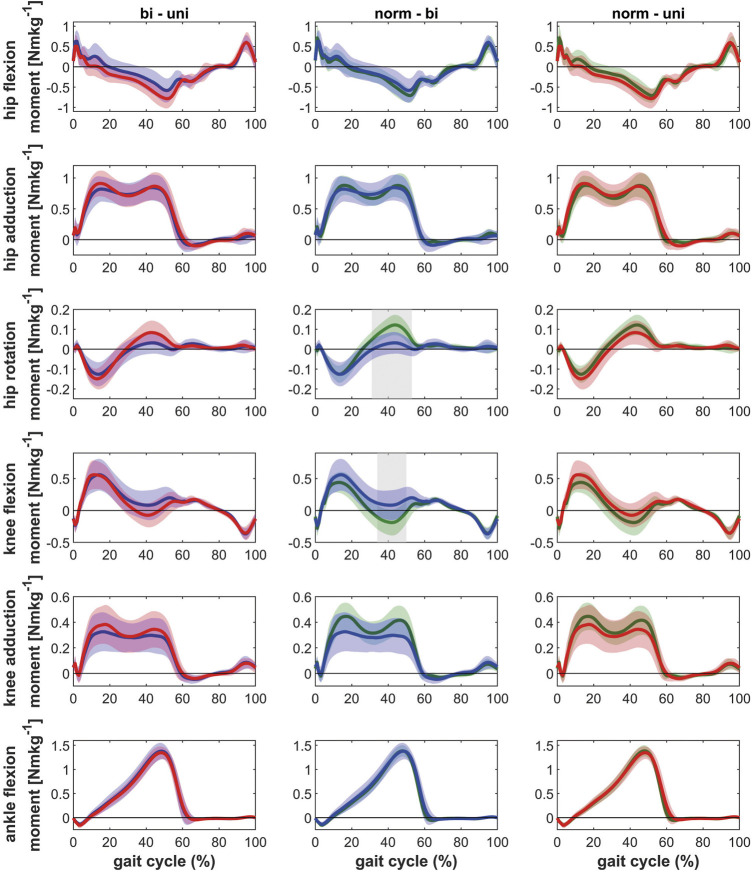
Comparison of the kinetics of the contralateral leg between bilateral hip OA patients, unilateral hip OA patients and healthy controls. Mean and one standard deviation of the external joint moments. Shaded areas indicate statistical differences. Blue lines = bilateral patients, red lines = unilateral patients, green lines = healthy controls. **(A–C)** hip flexion/extension moment: flexion (+)/extension (−); **(D–F)** hip adduction/abduction moment: adduction (+)/abduction (−); **(G–I)** hip rotation moment: internal rotation (+)/external rotation (−); **(J–L)** knee flexion/extension moment: flexion (+)/extension (−); **(M–O)** knee adduction/abduction moment: varus (+)/valgus (−); **(P–R)** ankle dorsiflexion/plantarflexion moment: dorsiflexion (+)/plantarflexion (−).

Regarding the kinetic parameters, it was found that the ipsilateral leg of patients with bilateral and unilateral hip OA had lower internal hip rotation moments, as well as a lower external knee adduction moments in the second half of stance compared to healthy controls (Figure 3 and Figure 4). On the ipsilateral side, bilateral patients additionally showed a lower hip extension moment compared to the healthy control group. Only the contralateral leg of bilateral patients showed differences compared to healthy controls: lower internal hip rotation moments and a reduced knee extension moment during the second half of stance.

**FIGURE 5:**
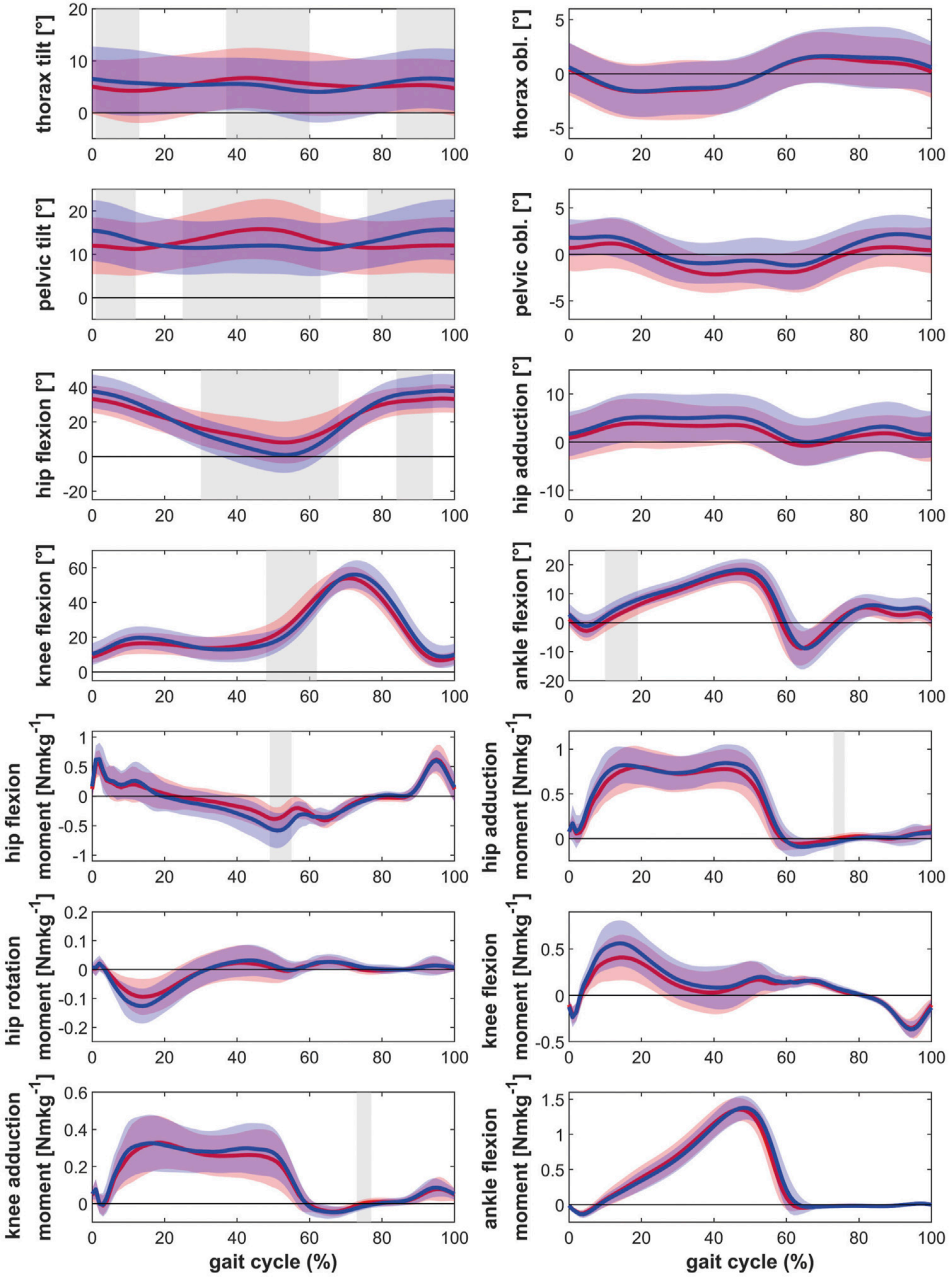
Comparison of the kinematics and kinetics between the ipsilateral and contralateral leg of bilateral hip OA patients. Mean and one standard deviation of the joint angles and the external joint moments. Shaded areas indicate statistical differences. Red lines = ipsilateral leg, blue lines = contralateral leg. **(A)** thorax tilt: anterior (+)/posterior (−); **(B)** thorax obliquity: towards the contralateral side (+)/towards the ipsilateral side (−); **(C)** pelvic tilt: anterior (+)/posterior (−); **(D)** pelvic obliquity: up (+)/down (−); **(E)** hip flexion/extension: flexion (+)/extension (−); **(F)** hip adduction/abduction: adduction (+)/abduction (−); **(G)** knee flexion/extension: flexion (+)/extension (−); **(H)** ankle dorsiflexion/plantarflexion: dorsiflexion (+)/plantarflexion (−); **(I)** hip flexion/extension moment: flexion (+)/extension (−); **(J)** hip adduction/abduction moment: adduction (+)/abduction (−); **(K)** hip rotation moment: internal rotation (+)/external rotation (−); **(L)** knee flexion/extension moment: flexion (+)/extension (−); **(M)** knee adduction/abduction moment: varus (+)/valgus (−); **(N)** ankle dorsiflexion/plantarflexion moment: dorsiflexion (+)/plantarflexion (−).

A closer look at both legs of the bilateral patients showed differences between the ipsilateral leg (leg to be operated on) and the contralateral leg (Figure 5). The most striking differences were the counteracting movements of the thorax and the pelvis in the sagittal plane (tilt) and the reduced hip extension at the end of the stance phase of the ipsilateral leg. The lower hip flexion at the beginning of stance and the lower hip extension at the end of stance defined the reduced hip range of motion in the ipsilateral leg. The reduced range of motion resulted in a slightly lower external hip extension moment in the second half of stance phase for the ipsilateral leg.

**FIGURE 6:**
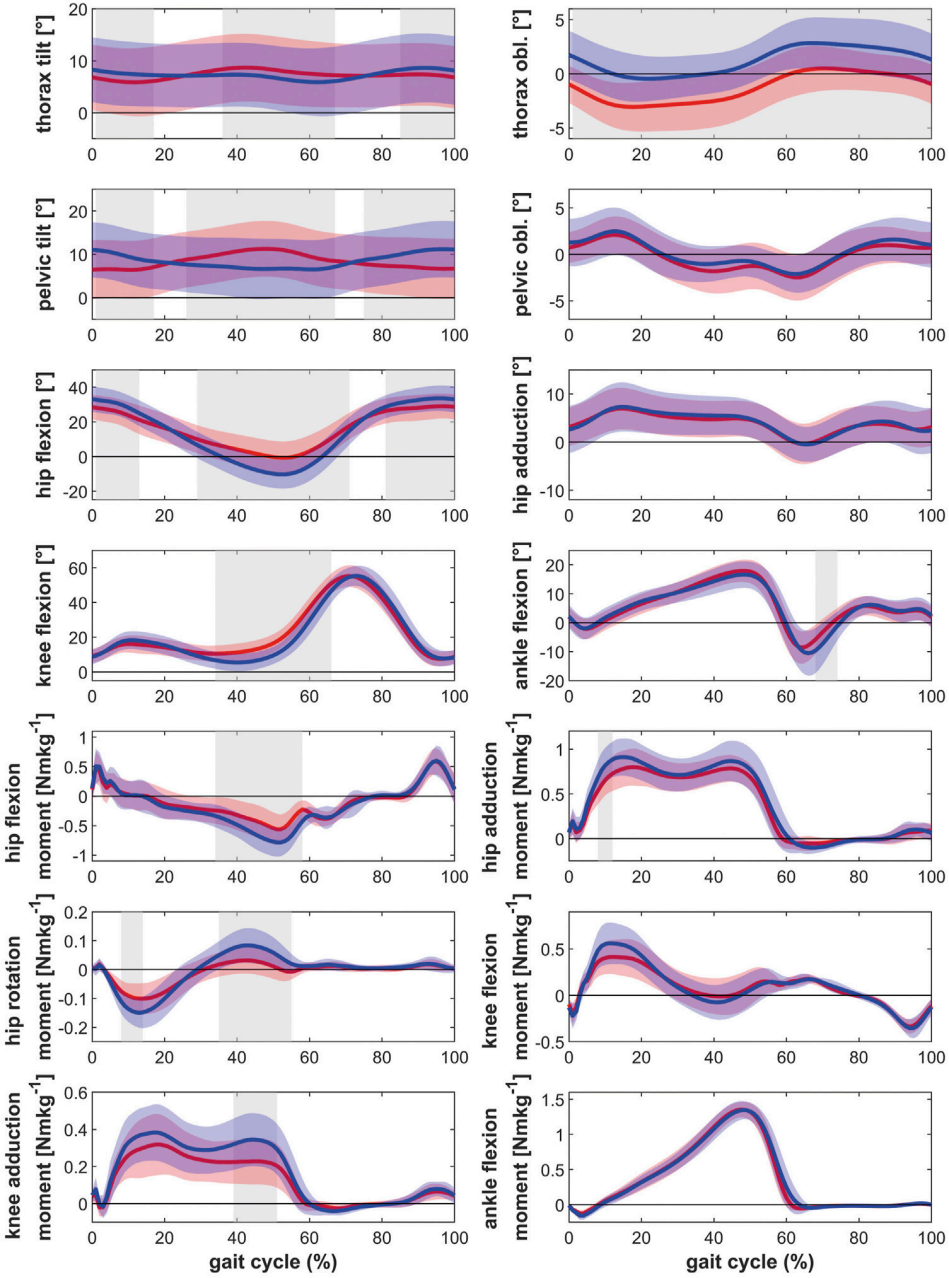
Comparison of the kinematics and kinetics between the ipsilateral and contralateral leg of unilateral hip OA patients. Mean and one standard deviation of the joint angles and the external joint moments. Shaded areas indicate statistical differences. Red lines = ipsilateral leg, blue lines = contralateral leg. **(A)** thorax tilt: anterior (+)/posterior (−); **(B)** thorax obliquity: towards the contralateral side (+)/towards the ipsilateral side (−); **(C)** pelvic tilt: anterior (+)/posterior (−); **(D)** pelvic obliquity: up (+)/down (−); **(E)** hip flexion/extension: flexion (+)/extension (−); **(F)** hip adduction/abduction: adduction (+)/abduction (−); **(G)** knee flexion/extension: flexion (+)/extension (−); **(H)** ankle dorsiflexion/plantarflexion: dorsiflexion (+)/plantarflexion (−); **(I)** hip flexion/extension moment: flexion (+)/extension (−); **(J)** hip adduction/abduction moment: adduction (+)/abduction (−); **(K)** hip rotation moment: internal rotation (+)/external rotation (−); **(L)** knee flexion/extension moment: flexion (+)/extension (−); **(M)** knee adduction/abduction moment: varus (+)/valgus (−); **(N)** ankle dorsiflexion/plantarflexion moment: dorsiflexion (+)/plantarflexion (−).

Besides the above-mentioned differences (thorax tilt, pelvic tilt, hip flexion-extension and hip flexion extension moment), unilateral patients had an asymmetric thorax obliquity (more thorax lean towards the ipsilateral side), higher hip adduction moments during the first half of stance and higher knee adduction moments during the second half of stance in the contralateral leg (Figure 6). The contralateral leg further showed higher rotation moments and more knee extension during the second half of stance compared to the ipsilateral leg.

### Symmetry

**FIGURE 7:**
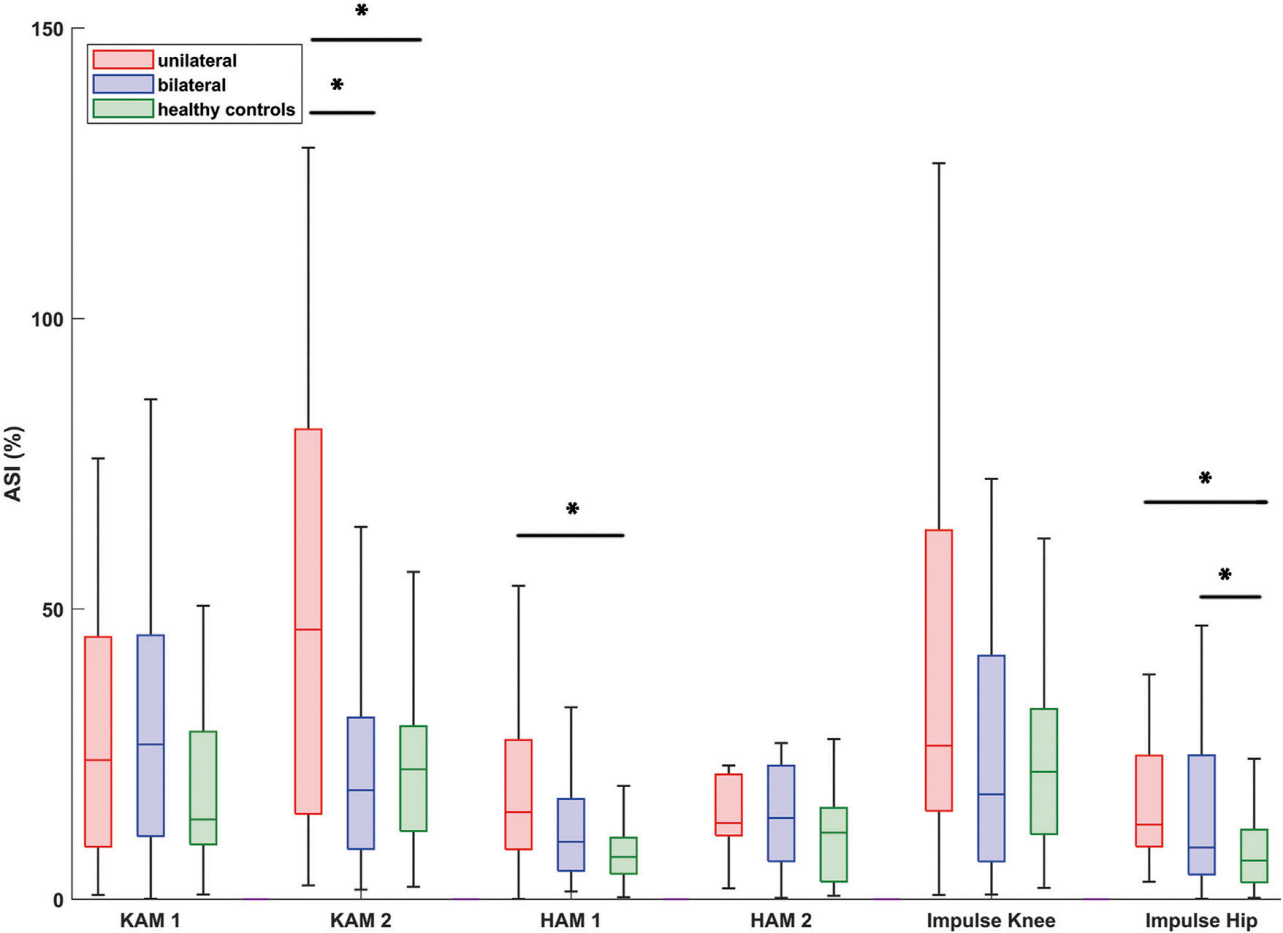
Box-and-whisker-plots of Absolute Symmetry Indices (ASI) of the external knee and hip joint moments as well as the knee and hip impulses. Boxes represent 50% of the data (interquartile range), the line within the box represents the median and each whisker represents 1.5 times the interquartile range. Significant differences between groups are indicated with an asterisk. Abbreviations: KAM_1/HAM_1: knee / hip adduction moment during the first half of stance; KAM_2 / HAM_2: knee / hip adduction moment during the second half of stance.

The symmetry values differed between bilateral patients and healthy controls only with regard to the hip moment impulse: healthy controls showed a higher gait symmetry compared to the bilateral patients (Figure 7). Unilateral patients showed a lower symmetry compared to healthy controls for the knee adduction moment during the second half of stance, the hip adduction moment during the first half of stance and the hip moment impulse. Only the symmetry in the knee adduction moment during the second half of stance differed significantly between the bilateral and unilateral patients, with a lower symmetry for unilateral patients.

## Discussion

The main aim of the present study was to investigate the gait pattern of bilateral hip OA patients. This research showed that, in contrast to unilateral patients, the gait pattern of both legs of bilateral patients deviated from matched healthy controls. Although patients with bilateral hip OA show more symmetrical frontal plane knee joint moments, a pathological external knee adduction moment in the second half of stance in the ipsilateral leg is present in patients with unilateral and bilateral hip OA.

### Kinematics

The present study showed that bilateral patients walk with a gait pattern, which has been previously published for unilateral patients: more thorax tilt and lean, more pelvic tilt, less hip extension and less knee extension (Shakoor et al., 2003; Foucher et al., 2007; van Drongelen et al., 2021). The above-mentioned results were found for both legs of the bilateral hip OA patients. Kubota et al. (Kubota et al., 2007) also studied bilateral patients. They found that bilateral patients walked with a forward tilted pelvis and a lean to the ipsilateral side during stance, as was found in the present work. In the current study, significant differences were found between the ipsi- and contralateral leg of bilateral affected OA patients. The patients showed an increased pelvic tilt and a decreased hip extension towards the end of the stance phase for the ipsilateral leg. The contralateral leg had to counter the pelvic movement and showed more hip extension. A comparison to the study of Kubota et al. (Kubota et al., 2007) was not possible, as from that study it did not become clear whether mean values for both legs or data for just one leg were analyzed.

In the present study, a significant higher BMI was found for the unilateral patients compared to the healthy controls. BMI has been associated with lower knee and hip range of motion (Holla et al., 2011) and could be responsible for the differences between the unilateral patients and healthy controls. However, knee and hip range of motion have also been associated with disability (Steultjens et al., 2000) and since no differences were found between bilateral and unilateral patients in BMI nor in the knee and hip flexion the differences in BMI were neglected.

### Kinetics

The second aim of the present study dealt with the magnitude and symmetry of the external knee and hip adduction moments. We hypothesized that, in contrast to patients with unilateral hip OA, the external knee and hip adduction moments of the contralateral side are not different compared to the ipsilateral side (more affected side) in bilateral affected patients with hip OA. As a result, bilateral hip OA patients show a more symmetrical joint load compared to unilateral hip OA patients.

For the hip, no differences in the external hip adduction moments were found between patients and healthy controls. This is in contrast to the findings of Foucher et al. (Foucher et al., 2007), who attributed the reduced hip adduction moments on the ipsilateral side in unilateral hip OA patients to an impairment of the hip abductor muscles. Also Kubota et al. (Kubota et al., 2007) reported lower external hip adduction moments. In that study, the reduction in the hip adduction moment was compensated with increased power generation in the ankle joint. However, the patients in the study of Kubota et al. (Kubota et al., 2007) had a very low walking speed (∼0.65 ms^−1^) so that the characteristic double peak was not visible in the hip adduction moment, which might indicate that patients with low functional status were studied (Bahl et al., 2020). The patients in the present study walked at a speed similar to unilateral hip OA patients reported in various studies (Foucher, 2017; Schmidt et al., 2017). In comparison to the healthy controls, the typical double peak in the knee and hip adduction moments seemed to be missing as well (Figures 3,4). However, in the direct comparison of the legs (Figure 5) and in the comparison to unilateral patients it can be seen that the double peak is less pronounced and the curve flattened in patients with bilateral hip OA compared to healthy controls.

Besides the external hip adduction moment, the external knee adduction moments are important parameters (which can be determined by gait analysis), as the peak knee adduction moment has been related to the initiation and progression of knee OA (Miyazaki et al., 2002; Andriacchi and Mündermann, 2006). Both the bilateral and unilateral patients showed a reduced knee adduction moment in the second half of stance phase in the ipsilateral leg, but not in the contralateral leg, compared to the healthy controls. Whereas the unilateral patients showed a clear higher knee adduction moment in the contralateral knee, the bilateral patients had similar knee adduction moments in both knees.

The ipsilateral trunk lean toward the affected stance limb, shown in our patients with unilateral and bilateral hip OA, has been identified as an important compensatory mechanism to unload the hip (Stief et al., 2014). This compensating strategy is also responsible for the redistribution of loads transferred through the medial and lateral knee compartment (Simic et al., 2012; Stief et al., 2014). Body weight and thus the ground reaction force vector is moved toward the center of the knee and hip joint, which reduces medial knee load in a dose-response relationship, with larger lean angles leading to greater reductions in the knee adduction moment (Simic et al., 2012). Mündermann et al. (Mündermann et al., 2008) and van den Noort et al. (van den Noort et al., 2013) have demonstrated that lateral trunk lean has the potential of reducing the knee adduction moment during walking by up to 65% in healthy subjects. Besides the unloading mechanism, ipsilateral trunk lean can shift the physiological external knee adduction moment into a pathological external abduction moment (valgus moment). This lateral adjustment is argued to increase the lateral tibio-femoral compartment load (Sharma et al., 2000). However, at the moment, it is still questionable whether the change in the medial-to-lateral knee joint load distribution in the ipsilateral leg leads to increased progression or even initiates degenerative changes of the lateral compartment of the knee, since lower medial loading does not necessarily indicate higher lateral loading (van Drongelen et al., 2020b). Various studies reported a higher risk for the development of OA in the contralateral knee joint after THA (Shakoor et al., 2003; Umeda et al., 2009). However, these studies only reported degenerative findings and did not specify the medial or lateral knee compartment. Gillam et al. (2013) stated that further studies are needed to investigate whether other factors besides load play a role as well. At the moment only Weidow et al. (Weidow et al., 2005) showed an increased probability of OA in the lateral knee compartment of the ipsilateral leg in patients with hip OA which could be attributed to the morphology of the hip and pelvis in the patient collective.

### Symmetry

We hypothesized that bilateral hip OA patients show a more symmetrical joint load compared to unilateral hip OA patients due to the symmetrical joint moments. Indeed, bilateral hip OA patients walked with more symmetrical knee and hip joint moments, almost similar to healthy controls. Compared to the healthy controls, the bilateral patients only showed lower symmetry in the hip moment impulse. Besides the asymmetry in the hip moment impulse, the unilateral patients also showed a lower symmetry for the hip adduction moment in the first half of stance and the knee adduction moment in the second half of stance compared to the healthy controls.

A closer look at the symmetry data showed that some patients were outside the whiskers, which represent 1.5 times the interquartile range (outliers not displayed in Figure 7). However, in general the gait symmetry of bilateral hip OA patients is comparable to the healthy controls. The present study confirmed the values presented by Schmidt et al. (Schmidt et al., 2017) who found clearly lower symmetry for unilateral hip OA patients compared to healthy controls. Schmidt et al. (Schmidt et al., 2017) reported more variation for unilateral hip OA patients with regard to ASI than healthy controls. Individual adopted gait patterns, which might be caused by pain, can explain the inter-subject variability.

With a more symmetrical load, one could argue that the overload on the contralateral leg is not present in bilateral patients, which could lower the risk for knee and hip OA in the contralateral leg. It must be noted that ASI does not give any qualitative information on which side is overloaded or underloaded. Further, the knee adduction moments in bilateral hip OA patients were lower in both legs compared to healthy controls (significant for the ipsilateral leg and not significant for the contralateral leg). As mentioned above, a lower knee adduction moment might indicate a shift of the load from the medial to the lateral knee compartment (Schmidt et al., 2017), which can facilitate degenerative changes of the lateral knee compartment.

A direct comparison of bilateral and unilateral hip OA patients is worth discussing. Usually, bilateral affected patients are excluded from studies on hip OA, which reduces the sample size considerably. From the present study, it became clear that there were no differences in the kinematics and kinetics of the ipsilateral leg between unilateral and bilateral hip OA patients. As such, it can be concluded that the ipsilateral leg of the bilateral patients could be included in studies next to unilateral hip OA patients. In contrast, and based on the larger hip range of motion, the contralateral leg of bilateral patients is not automatically suited for inclusion in studies like the ipsilateral leg.

### Limitations

External knee and hip adduction moments were chosen as the primary outcome, as they are still the most commonly used measures of joint loading with an important relationship to disease progression and initiation (Hurwitz et al., 2001; Miyazaki et al., 2002; Andriacchi and Mündermann, 2006; Lenaerts et al., 2009; Wesseling et al., 2015; Tateuchi et al., 2017). At the moment it is still questionable whether the change in medial-to-lateral knee joint load distribution in the ipsilateral leg leads to increased progression or even initiates degenerative changes of the lateral compartment of the knee, since lower medial loading does not necessarily indicate higher lateral loading (Andriacchi, 2013). Alternative measures of joint load, including compressive force in the medial and lateral knee compartment measured via by musculoskeletal modeling including muscle activity and forces (Holder et al., 2020), might be able to answer this question in the future.

## Conclusion

This research showed that, when corrected for speed, the gait kinematics and kinetics of both legs of patients with bilateral hip OA differ from healthy controls. The ipsilateral leg of the bilateral patients could be included in studies in addition to unilateral hip OA patients as no differences were found. Bilateral patients have a symmetrical knee joint loading, in contrast to the asymmetrical knee joint loading in unilateral hip OA patients. Although patients with bilateral hip OA show more symmetrical frontal plane knee joint moments, a pathological external knee adduction moment in the second half of stance in the ipsilateral leg is present in both patients with unilateral and bilateral hip OA.

## Data Availability

The raw data supporting the conclusion of this article will be made available by the authors, without undue reservation.

## Abbreviations

OA, osteoarthritis; THA, total hip arthroplasty; SPM, Statistical Parametric Mapping
