# Supplementary material for: Comparison of Gait Symmetry and Joint Moments in Unilateral and Bilateral Hip Osteoarthritis Patients and Healthy Controls
Source: Front Bioeng Biotechnol. 2021 Nov 4;9:756460. doi: 10.3389/fbioe.2021.756460 (PMC8599579; doi:10.3389/fbioe.2021.756460)
Supplement: Supplementary file 1 [file DataSheet1.DOCX]

##
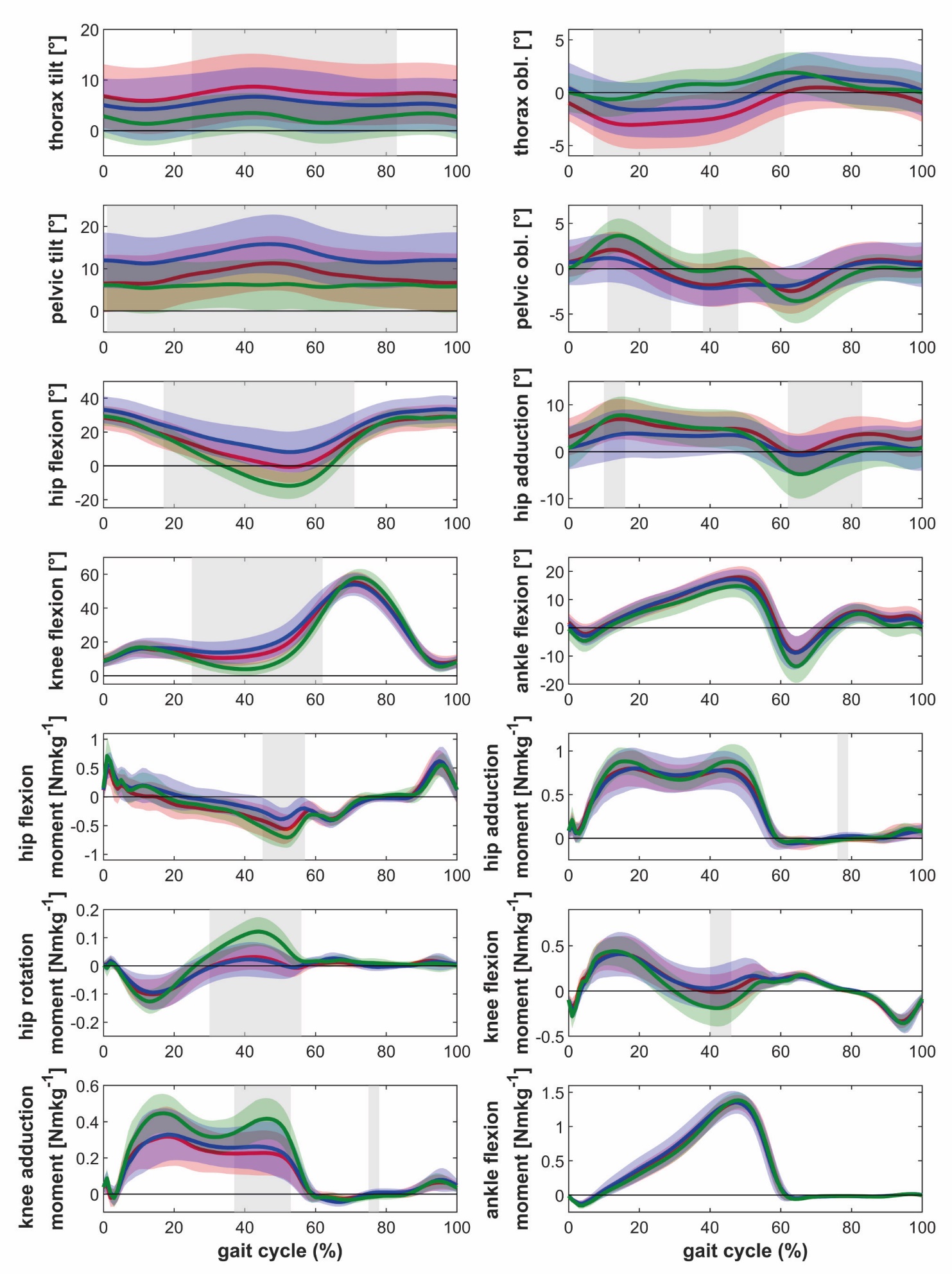


**Supplementary Figure 1.** **Comparison of the kinematics and kinetics of the ipsilateral leg between bilateral hip OA patients, unilateral hip OA patients and healthy controls**

Mean and one standard deviation of the joint angles and the external joint moments. Shaded areas indicate statistical differences from the ANOVA. Blue lines = bilateral patients, red lines = unilateral patients, green lines= healthy controls.

(A) Thorax tilt: anterior (+) / posterior (-); (B) thorax obliquity: towards the contralateral side (+) / towards the ipsilateral side (−); (C) pelvic tilt: anterior (+) / posterior (-); (D) pelvic obliquity: up (+) / down (-); (E) hip flexion/extension: flexion (+) / extension (−); (F) hip adduction/abduction: adduction (+) / abduction (-); (G) knee flexion/extension: flexion (+) / extension (−); (H) ankle dorsiflexion/plantarflexion: dorsiflexion (+) / plantarflexion (-); (I) hip flexion/extension moment: flexion (+) / extension (-); (J) hip adduction/abduction moment: adduction (+) / abduction (-); (K) hip rotation moment: internal rotation (+) / external rotation (-); (L) knee flexion/extension moment: flexion (+) / extension (-); (M) knee adduction/abduction moment: varus (+) / valgus (-); (N) ankle dorsiflexion/plantarflexion moment: dorsiflexion (+) / plantarflexion (-).

**
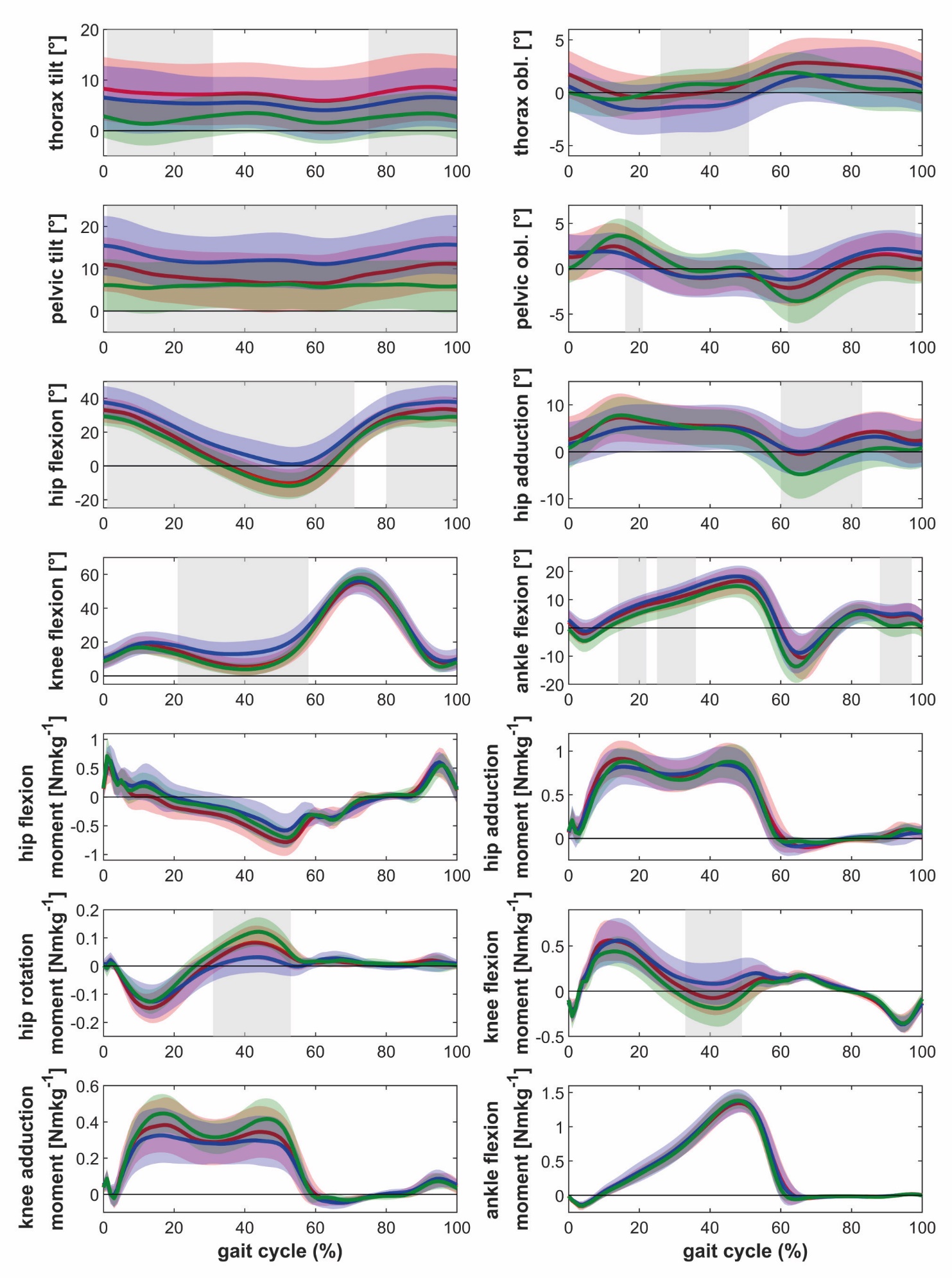
**

**Supplementary Figure 2.** **Comparison of the kinematics and kinetics of the contralateral leg between bilateral hip OA patients, unilateral hip OA patients and healthy controls**

Mean and one standard deviation of the joint angles and the external joint moments. Shaded areas indicate statistical differences from the ANOVA. Blue lines = bilateral patients, red lines = unilateral patients, green lines= healthy controls.

(A) Thorax tilt: anterior (+) / posterior (-); (B) thorax obliquity: towards the contralateral side (+) / towards the ipsilateral side (−); (C) pelvic tilt: anterior (+) / posterior (-); (D) pelvic obliquity: up (+) / down (-); (E) hip flexion/extension: flexion (+) / extension (−); (F) hip adduction/abduction: adduction (+) / abduction (-); (G) knee flexion/extension: flexion (+) / extension (−); (H) ankle dorsiflexion/plantarflexion: dorsiflexion (+) / plantarflexion (-); (I) hip flexion/extension moment: flexion (+) / extension (-); (J) hip adduction/abduction moment: adduction (+) / abduction (-); (K) hip rotation moment: internal rotation (+) / external rotation (-); (L) knee flexion/extension moment: flexion (+) / extension (-); (M) knee adduction/abduction moment: varus (+) / valgus (-); (N) ankle dorsiflexion/plantarflexion moment: dorsiflexion (+) / plantarflexion (-).
